# Competition mode and soil nutrient status shape the role of soil microbes in the diversity–invasibility relationship

**DOI:** 10.1002/ece3.11425

**Published:** 2024-05-14

**Authors:** Haokun Li, Xinyu Hu, Xinze Geng, Bo Xiao, Wei Miao, Zhiguang Xu, Yizhuo Deng, Bohan Jiang, Yuping Hou

**Affiliations:** ^1^ College of Life Sciences Ludong University Yantai China; ^2^ Analysis and Testing Center Ludong University Yantai China; ^3^ Kuyushan Forest Farm Yantai China

**Keywords:** competition mode, invasibility, nutrient status, plant diversity, plant invasion, soil microorganisms

## Abstract

Understanding the relationship between plant diversity and invasibility is essential in invasion ecology. Species‐rich communities are hypothesized to be more resistant to invasions than species‐poor communities. However, while soil microorganisms play a crucial role in regulating this diversity–invasibility relationship, the effects of plant competition mode and soil nutrient status on their role remain unclear. To address this, we conducted a two‐stage greenhouse experiment. Soils were first conditioned by growing nine native species separately in them for 1 year, then mixed in various configurations with soils conditioned using one, three, or six species, respectively. Next, we inoculated the mixed soil into sterilized substrate soil and planted the alien species *Rhus typhina* and native species *Ailanthus altissima* as test plants. We set up two competition modes (intraspecific and interspecific) and two nutrient levels (fertilization using slow‐release fertilizer and nonfertilization). Under intraspecific competition, regardless of fertilization, the biomass of the alien species was higher in soil conditioned by six native species. By contrast, under interspecific competition, the biomass increased without fertilization but remained stable with fertilization in soil conditioned by six native species. Analysis of soil microbes suggests that pathogens and symbiotic fungi in diverse plant communities influenced *R. typhina* growth, which varied with competition mode and nutrient status. Our findings suggest that the soil microbiome is pivotal in mediating the diversity–invasibility relationship, and this influence varies according to competition mode and nutrient status.

## Graphical abstract

Soil microbiome is pivotal in mediating the diversity–invasibility relationship, and this influence varies with competition mode and nutrient status.

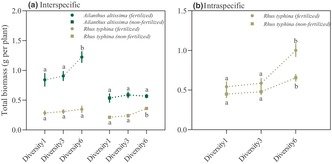

## 1 INTRODUCTION

Globalization is accelerating the invasion of landscapes by alien species (Seebens et al., 2017). The success of alien species invasion depends not only on the invasiveness of the invading species but also on the invasibility of the invaded communities (Dai et al., 2020). Elton hypothesized that species‐rich communities exhibit greater resistance to invasion by alien species (Elton, 1958). However, studies of this hypothesis have yielded contradictory results (Ernst et al., 2021; Fridley et al., 2007; Smith & Côté, 2019), indicating both positive and negative relationships between species richness and invasibility. This indicates that the negative association between plant diversity and invasibility does not exist in all ecosystems (Davies et al., 2005; Herben, 2005; Richardson & Pyšek, 2006).

Plant communities with different levels of species richness can shape the soil microbial community structure (Lodge, 1997; Waldrop et al., 2006). Soil microbes include different functional groups that can promote (as mutualistic symbionts) or reduce (as pathogens) plant growth (Morris et al., 2007). Therefore, plant diversity‐mediated changes in soil microbial communities may affect colonization or invasion by alien species (Crawford et al., 2019; Hart et al., 2003; Mallon et al., 2015; Wang et al., 2022; Zhang et al., 2020). Various hypotheses have been proposed regarding the effects of microbes on diversity–invasibility relationships. The “amplification effect” hypothesis proposes that plant species‐rich communities harbor a greater diversity and abundance of pathogens (Hudson et al., 2006; Keesing et al., 2006), increasing the likelihood of these pathogens negatively affecting alien plants. By contrast, the “dilution effect” hypothesis proposes that species‐rich communities exhibit a lower abundance of highly susceptible hosts (Ostfeld & Keesing, 2012; Schmidt & Ostfeld, 2001), reducing pathogen prevalence and the negative impact on alien plants. Following these arguments, mutualism can lead to similar or opposing hypotheses. First, if plant diversity enhances mutualistic symbiosis, species‐rich communities are more likely to include mutualistic symbionts that benefit alien plants; these communities will thus be less able to resist alien species (Reinhart & Callaway, 2006). Second, if plant diversity dilutes the prevalence of mutualistic symbiosis, species‐rich communities will be better able to resist alien plants. Soil fungi, which include many mutualistic and pathogenic species, could therefore play significant roles in influencing the diversity–invasibility relationship. Therefore, it is necessary to further investigate the influence of soil fungi on this relationship. However, little is known about how plant diversity affects alien plant invasion by driving changes in soil fungal communities.

Environmental factors such as competition and nutrient variability can alter the role of soil microorganisms and the directionality of their effect (Islam et al., 2020). The competition mode can alter interactions between microorganisms and plants (Abbott et al., 2015; Kandlikar et al., 2019; Stein & Mangan, 2020). Under interspecific competition, soil microbes can increase plant resistance to environmental stress, whereas intraspecific competition does not have this effect (Fitzpatrick et al., 2019). Compared with intraspecific competition, during interspecific competition, invasive Asteraceae species can promote invasion by increasing the rate of colonization by arbuscular mycorrhizal fungi [AMF] (Sun et al., 2022). Similarly, nutrient variability can affect the interactions between soil microorganisms and plants (Johnson et al., 1997; Kaur et al., 2022; Yang et al., 2016). Under low‐phosphorus conditions, AMF enhance the competitive advantage of alien plants over native plants, whereas under high‐phosphorus conditions, they reduced this advantage (Chen et al., 2019). The effects of AMF on plants range from mutualism to parasitism and are strongly influenced by soil nutrient availability (Johnson, 2010). These studies reveal that competition mode and nutrient level can affect the structure and function of communities via microbial pathways. However, it remains unclear whether competition mode and nutrient level affect the role of microorganisms in the diversity–invasibility relationship.

To address this, we aimed to simulate various levels of plant diversity within a temperate forest community context to examine the impact of soil microorganisms on community invasibility, and how competition mode and nutrient level modify this relationship. We conditioned the soil by growing nine independent native species for 1 year. By mixing these soil samples in different combinations, we obtained soil samples conditioned under three simulated levels of plant community diversity (Diversity1, Diversity3, and Diversity6). The alien species *Rhus typhina* and native co‐occurring species *Ailanthus altissima* were planted in these soil mixtures under intraspecific or interspecific competition, and with or without slow‐release fertilizer treatment. The main research questions were as follows: (1) What is the role of soil microorganisms in the diversity–invasibility relationship in forest ecosystems? (2) Do competition mode and nutrient level influence the role of soil microorganisms in the diversity–invasibility relationship?

## 2 MATERIALS AND METHODS

### 2.1 Study site and species

In our greenhouse experiment, we grew nine plant species native to China (Table S1), including *Indigofera kirilowii*, *Rhus chinensis*, and *Quercus acutissima* among others, in separate pots. These species are commonly found in the eastern region of the Shandong Peninsula, where they have overlapping distributions, suggesting their natural coexistence (Wang & Zhou, 2000). We used a pair of deciduous shrubs as test species for our study: *R. typhina*, which is native to North America and invasive in northern China, and *A. altissima*, which is native to East Asia but invasive in North America (Sladonja et al., 2015). Both species grow rapidly and are commonly used in restoration projects in northern China (Guo & Xu, 2019). *Rhus typhina* was introduced as an ornamental plant in China in 1959 and spread rapidly in various habitats, including shrub grasslands and secondary forests (Wang et al., 2008). *Ailanthus altissima*, shares a similar ecological niche with *R. typhina* and often coexists with it in natural settings (Petruzzellis et al., 2019). For the experiment, seeds were collected from the Kunyu Mountain National Nature Reserve, eastern Shandong Peninsula (121.37–121.48°E, 37.10–37.19°N). This 154.165 km^2^ reserve features a temperate monsoon and maritime climate, predominantly brown loam soil, and is part of the warm temperate deciduous broad‐leaved forest biome. Notably, it contains the largest and most well‐preserved natural ecosystem of Japanese red pine (*Pinus densiflora*) in China (and possibly worldwide), contributing to its rich biodiversity (Yu, 2009). The reserve also supports a range of indigenous trees and shrubs including *Q. acutissima*, *Lespedeza bicolor*, and *I. kirilowii* (Wang & Zhou, 2000).

### 2.2 Experimental setup

**FIGURE 1:**
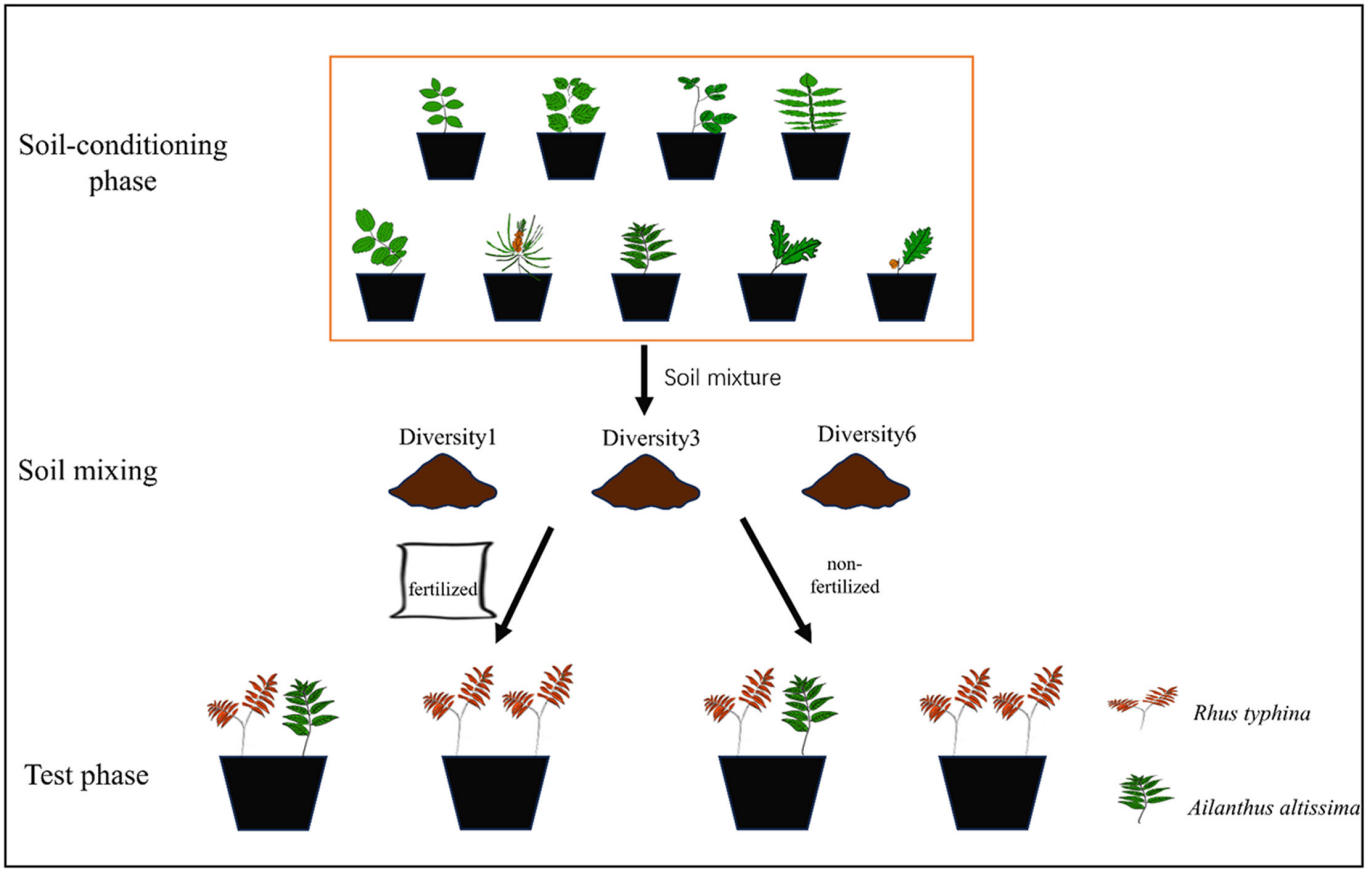
Experimental scheme involving three steps. Step 1: soil‐conditioning phase. To test the effects of native plant diversity on alien plants, soil samples were collected from pots in which nine native plants were grown separately. Step 2: soil‐mixing phase. Samples of the conditioned soils were mixed in various configurations to establish three diversity levels: no mixing (one species), three species mixed, or six species mixed. Step 3: testing phase. The mixed soil (25 mL) was inoculated onto sterilized substrate soil (500 mL per pot). To establish the two modes of competition (intraspecific and interspecific), we planted individuals of the alien *Rhus typhina* and native *Ailanthus altissima* and manipulated the nutrient level by providing fertilization or no fertilization.

The experimental scheme, which involved three steps, is shown in Figure 1. In step 1, the soil conditioning stage, we conditioned the soil for a year by planting the nine native species separately in the same soil. In step 2, the soil‐mixing stage, we collected soil samples from the soil‐conditioning pots and mixed them to create mixtures of soil reflecting conditioning by one, three, or six species. In step 3, the testing stage, we inoculated each soil mixture onto sterilized substrate soil, set up two competition modes (intraspecific vs. interspecific), and simulated two nutrient levels (fertilization with a slow‐release fertilizer vs. nonfertilization). The entire plant biomass was then harvested.

#### 2.2.1 Soil‐conditioning phase

On September 1, 2021, seeds of the nine native species were sown separately in a tray (109 × 109 cm) filled with sterilized vermiculite. These trays were placed in a greenhouse at temperatures between 18 and 25°C under natural light. Concurrently, we collected soil from an open area around the greenhouse and removed plant roots and large pieces of debris using a 1 cm sieve. For each native species, we selected 15 similarly grown seedlings and transplanted one seedling into one‐gallon pots that were filled with a mixture of 25% open‐area soil, 37.5% river sand, and 37.5% vermiculite. This resulted in 135 pots. To maintain humidity and prevent cross‐contamination from solution runoff, the pots were placed in pot trays. Seedlings that died within 2 weeks of transplantation were immediately replaced. The pots were watered as needed and were randomly rearranged every 2 weeks. On August 15, 2022, we removed all the aboveground plant parts, used a 5 mm sieve to remove fine roots and other impurities from the soil, and then mixed the soil from all pots for each species, obtaining nine soil samples.

#### 2.2.2 Soil‐mixing phase

To create the species‐diversity gradient, we mixed soil samples from the soil‐conditioning phase to create three diversity levels (Diversity1, Diversity3, and Diversity6; Figure 1). In the soil‐conditioning phase, there were nine native species, resulting in nine possible soil samples for Diversity1 and 84 potential combinations for Diversity3 and Diversity6. To equalize the number of combinations for each diversity treatment, we randomly selected nine combinations each from the sets of Diversity3 and Diversity6 combinations, obtaining 27 treatments (Table S2). The study design followed that of Zhang et al. (2020), with a randomized selection of combinations to ensure equal representation and frequency of each species across treatments.

#### 2.2.3 Testing phase

On August 15, 2022, we sowed seeds of *A. altissima* and *R. typhina* in trays filled with sterilized nutrient soil and perlite. At the same time, we collected 25 mL soil samples (~5% of the soil volume; Fahey & Flory, 2021) from each type of soil mixture and inoculated them into pots with a capacity of 700 mL. These pots were filled with 500 mL of substrate soil (open‐area soil: river sand, 1:1) that had been sterilized using high‐temperature steam (121°C, 0.105 MPa, 30 min) once a day for 3 days. These pots were then left in a greenhouse for 2 weeks. On September 1, 2022, we selected *A. altissima* and *R. typhina* seedlings at similar growth states and transplanted them into the pots, which were divided into a fertilizer group (treated with a universal slow‐release fertilizer, N:P:K, 20%:20%:10%) and a nonfertilized group. Under both nutrient levels, 135 pots were planted with two seedlings of *R. typhina* for the intraspecific competition mode; another 135 pots were planted with one seedling of *A. altissima* and one of *R. typhina* for the interspecific competition mode. Each treatment was replicated five times. In total, 540 pots were used (3 diversity categories × 9 species combinations × 2 nutrient levels × 2 competition modes × 5 replicates = 540 pots). During cultivation, we provided natural light and a sufficient water supply, regularly removed all weeds from the pots, and randomly adjusted the position of the pots every 2 weeks to avoid positional effects.

At 8 weeks after the start of the test phase, all aboveground and belowground parts of the test plants were harvested and rinsed with water. Owing to the death of some seedlings during the growth period, 502 *R. typhina* individuals (interspecific: 239, intraspecific: 263) and 259 *A. altissima* individuals were harvested. The collected plant tissue was placed in a 70°C oven to dry for a week and was then weighed. The biomass of two *R. typhina* plants was measured together under intraspecific competition, and the biomass of *R. typhina* and *A. altissima* were measured separately under interspecific competition.

### 2.3 Soil sampling, DNA extraction, amplicon sequencing, and bioinformatics analysis

The 27 mixed fresh soil samples, collected from various diversity treatments and subsequently stored in a refrigerator at −80°C, were analyzed for soil fungal community composition. The total microbial community DNA was extracted according to the instructions of the E.Z.N.A. Soil DNA Kit (Omega Bio‐tek, Norcross, GA). The quality of the extracted DNA was verified using 1% agarose gel electrophoresis, and DNA concentration and purity were measured using a NanoDrop 2000 device (Thermo Fisher Scientific, Waltham, MA). The fungal rRNA internal transcribed spacer (ITS) region was amplified using the primer set ITS1F (5′‐CTTGGTCATTTAGAGGAAGTAA‐3′) and ITS2R (5′‐GCTGCGTTCTTCATCGATGC‐3′). Sequencing was performed using a MiSeq PE300/NovaSeq PE250 platform (Illumina, San Diego, CA). The Illumina MiSeq platform has a higher throughput and lower error rate than other high‐throughput sequencers (Frey et al., 2014; Loman et al., 2012).

Fungal sequences were classified using the UNITE database (version 8.0) and using the USEARCH11‐uparse algorithm for clustering. Operational taxonomic unit (OTU) sequence similarity was 0.97. Species classification was performed using the unite 8.0/its fungi database, and classification confidence was 0.7. As fungal DNA extraction failed for one sample, we obtained 26 samples of ITS rDNA.

### 2.4 Statistical analysis

To facilitate the comparison of *R. typhina* biomass under different competition modes, the total biomass of two *R. typhina* in intraspecific competition was first calculated. Afterward, the value was divided by the number of plants to obtain the average biomass per *R. typhina* plant. All subsequent analyses of *R. typhina* biomass under intraspecific competition are based on these average values. To investigate the effects of plant diversity, competition mode, and nutrient level, along with their interactions, on the total biomass of the alien species *R. typhina*, we employed a linear mixed model using the lme4 package (Bates et al., 2014) in R 4.0.5 (R Core Team, 2021). The total *R. typhina* biomass was square root‐transformed to enhance the normality and homoscedasticity of the residuals. The model included plant diversity, competition mode, and nutrient level as fixed factors, with *R. typhina* biomass as the response variable. Similarly, we analyzed the total biomass of *A. altissima* using a linear mixed model. Plant diversity and nutrient level were treated as fixed factors, and followed a square root transformation of *A. altissima* biomass to enhance normality and homoscedasticity. The species composition of native plant communities was used as random effects in all models. We then conducted pairwise comparisons of the three plant diversity levels on *R. typhina* and *A. altissima* biomass across both nutrient levels and competition modes using the multcomp package (Hothorn et al., 2008).

The soil microbial communities were analyzed using the Majorbio Bio‐Pharm platform (www.majorbio.com). Principal component analysis (PCA) and nonmetric multidimensional scaling (NMDS) were used to analyze β‐diversity and assess changes in soil fungal community structure under different plant diversity treatments. One‐way analysis of variance (ANOVA) and false discovery rate (FDR) correction were used to assess the impact of plant diversity on α‐diversity, fungal community function, and the relative abundance of major phyla and genera. The FUNGuild predictive tool (Nguyen et al., 2016) was used to predict fungal community function in soil samples. The differences in the proportions of plant pathogen and AMF across different levels of plant diversity were analyzed further using one‐way ANOVA. Linear regressions were performed to further explore the relationships between the diversity and abundance of major functional groups of soil fungi and the biomass of *R. typhina* and *A. altissima*.

## 3 RESULTS

### 3.1 Effects of plant diversity level, competition mode, and nutrient level on plant biomass

**FIGURE 2:**
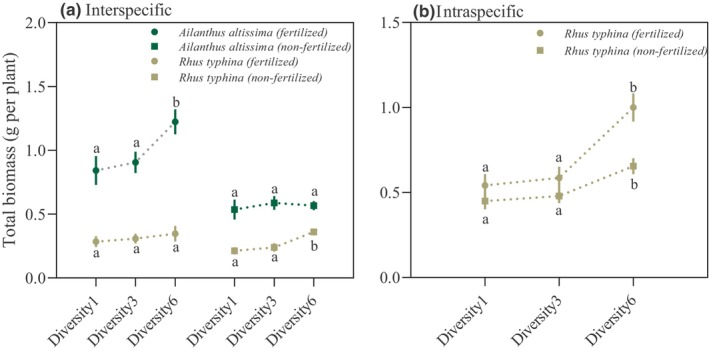
(a) Under interspecific competition, *Rhus typhina* and *Ailanthus altissima* biomass varies across different nutrient levels. (b) Under intraspecific competition, *R. typhina* biomass varies across different nutrient levels. Data are presented as mean (±SE). Letters identify diversity treatments that differ significantly at *p* < .05.

*Rhus typhina* growth was influenced significantly by plant diversity level, competition mode, and nutrient level (Table 1). High plant community diversity, intraspecific competition (as opposed to interspecific competition), and fertilization each increased *R. typhina* biomass significantly (Figure 2a,b; Table 1). Marginally significant interactions were observed between nutrient level and competition mode, competition mode and diversity levels, and among all three factors (Table 1). Specifically, when *R. typhina* was grown in interspecific competition without fertilization, *R. typhina* biomass was significantly higher for Diversity6 than for the other two diversity treatments, whereas with fertilization, it did not differ significantly among the plant diversity treatments (Figure 2a). By contrast, when in intraspecific competition, regardless of fertilization, *R. typhina* biomass was significantly higher under Diversity6 treatment than under the other two treatments (Figure 2b).

**TABLE 1:** Effects of plant diversity (Div) treatment, competition mode (Comp), nutrient level (Nutr), and their interactive effects on square root‐transformed total *Rhus typhina* biomass.

Factors/interactions	Total biomass of Rhus typhina (square root‐transformed)		
	*F*	*df*	*p*
Div	9.429	2	<.01
Comp	236.9	1	<.01
Nutr	15.94	1	<.01
Comp × Nutr	3.063	1	.080
Div × Nutr	0.021	2	.97
Div × Comp	2.837	2	.059
Div × Nutr × Comp	2.362	2	.095

*Note*: Differences are considered significant at *p* < .05 and marginally significant at *p* < .1.

*Ailanthus altissima* growth was influenced significantly by nutrient level (Table S3), and fertilization increased *A. altissima* biomass significantly (Figure 2a; Table S3). Significant interactions were observed between nutrient levels and diversity levels (Table S3). Specifically, without fertilization, *A. altissima* biomass was consistent among the Diversity1, Diversity3, and Diversity6 treatments; however, with fertilization, it was significantly higher under the Diversity6 treatment than under the Diversity1 and Diversity3 treatments (Figure 2a).

### 3.2 Fungal community of the conditioned soil

**FIGURE 3:**
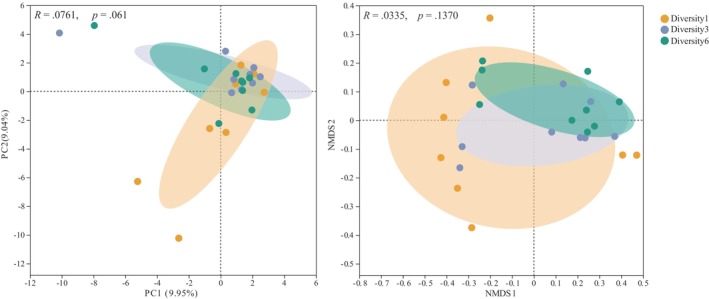
Principal component analysis (PCA) and nonmetric multidimensional scaling (NMDS) analysis of fungal community composition.

**FIGURE 4:**
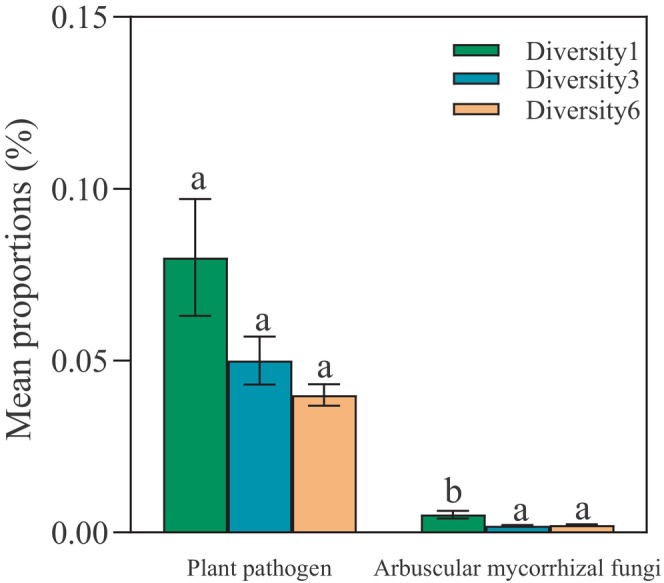
Relative abundance of the soil fungal community in soils conditioned using different levels of plant diversity, including the relative abundance of plant pathogens and arbuscular mycorrhizal fungi. Different letters indicate significant differences at *p* < .05 across plant diversity levels.

PCoA and NMDS revealed no differences in β‐diversity of the fungal community with an increase in plant diversity (Figure 3). The relative abundances of the dominant fungal phyla and genera did not vary significantly among the plant diversity levels (Figure S1). In addition, the Shannon index of the fungal community did not change (Table 2). FUNGuild results indicate no significant changes in the Shannon index of symbiotic fungi, pathogens, and plant pathogens; however, the AMF Shannon index increased with an increase in plant diversity (Table 2). As plant diversity increases, the relative abundance of AMF significantly decreases, and plant pathogen abundance shows a decreasing trend (Figure 4).

**TABLE 2:** Soil fungal community Shannon indices in soils conditioned at different plant diversity levels.

Community/Shannon index	All fungi	Pathotroph	Symbiotroph	Plant pathogen	Arbuscular mycorrhizal fungi
Diversity1	3.69	1.88	0.288	2.46	0.85 b
Diversity3	3.41	1.72	0.463	2.45	1.10 ab
Diversity6	3.35	1.73	0.393	2.53	1.35 a

*Note*: Different letters indicate significant differences at *p* < .05 across plant diversity levels.

### 3.3 Association between soil fungal community and plant biomass

**FIGURE 5:**
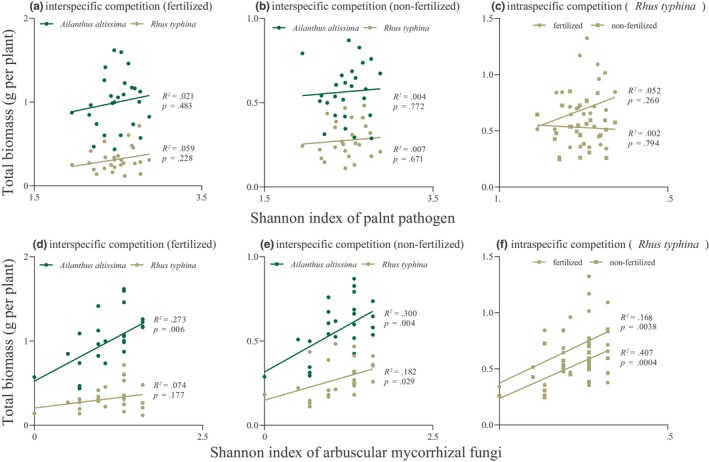
Relative abundance of arbuscular mycorrhizal fungi and plant pathogens in relation to total biomass per plant for *Rhus typhina* and *Ailanthus altissima* under different competition modes (interspecific and intraspecific) and nutrient levels (fertilized and nonfertilized). (a–c) Relative abundance of plant pathogens and total biomass per plant for *R. typhina* and *A. altissima*. (d–f) Relative abundance of arbuscular mycorrhizal fungi and total biomass per plant for *R. typhina* and *A. altissima*.

For *R. typhina*, under intraspecific competition, regardless of fertilization status, the Shannon diversity index of AMF was significantly associated with total *R. typhina* biomass (Figure 5f); these factors remained significantly associated under interspecific competition without fertilization, but not with fertilization (Figure 5d,e). No significant correlations were observed between the relative abundance of AMF (Figure S2d–f), Shannon diversity index (Figure 5a–c), or relative abundance of plant pathogens (Figure S2a–c) and the biomass of *R. typhina*. For *A. altissima*, under interspecific competition, the AMF Shannon diversity index was significantly associated with total biomass, regardless of fertilization status (Figure 5d,e). No significant correlations were observed between the relative abundance of AMF (Figure S2d–f), Shannon diversity index (Figure 5a–c), or relative abundance of plant pathogens (Figure S2a–c) and the biomass of *A. altissima*.

## 4 DISCUSSION

The diversity–invasibility hypothesis, proposed 60 years ago, has long been questioned. Here, we examined the effects of competition mode and nutrient status on the role of microorganisms in the relationship using species from a temperate forest community in China. Under intraspecific competition, regardless of fertilization, changes in soil microbial composition caused by the high‐diversity plant community enhanced *R. typhina* growth, whereas under interspecific competition, fertilization negated the enhancement. Therefore, although many prior studies have attributed the relationship between plant diversity and invasibility to resource competition (Byers & Noonburg, 2003), the findings of the present study suggest that the relationship may also be mediated by soil microbiota and influenced by competition mode and nutrient status.

According to the dilution effect hypothesis, in plant communities with high species diversity, the probability of hosts being infected by pathogens decreases (Ostfeld & Keesing, 2012; Schmidt & Ostfeld, 2001), leading to a reduction in pathogen abundance. Although the hypothesis focuses mainly on pathogens, it may also apply to symbiotic fungi, including AMF (Bever et al., 1997; Liao et al., 2015; Richardson et al., 2000). Our research supports the viewpoint: with an increase in plant diversity, we observed a decrease in the relative abundance of plant pathogens; moreover, AMF relative abundance also decreased significantly. These findings are consistent with our expectations from the dilution effect perspective regarding symbiotic fungi. The results of the present study demonstrate that increasing plant diversity can effectively reduce both pathogen and AMF relative abundance, potentially impacting alien plant growth and their competition with native plants.

Although alien plants may escape from their co‐evolved specialist pathogens (Mitchell & Power, 2003), they can still encounter resistance from generalist pathogens (Zhang et al., 2018). Although we did not distinguished between specialist and generalist pathogens directly, a general decrease in pathogen abundance might benefit all types of plants (Wang, Burrill, et al., 2023), including alien species (Barry et al., 2019; HilleRisLambers et al., 2012), helping to explain why *R. typhina* can grow better in communities with higher species diversity. Another potential explanation is that in plant communities with higher species diversity, a greater diversity of AMF was observed. The increase in AMF diversity makes it more likely for species closely associated with *R. typhina* to emerge (Dong et al., 2021; Lamit et al., 2022), thereby facilitating *R. typhina* growth. Notably, our observations of the soil fungal community were conducted before the start of the testing phase, and this soil fungal community structure could change during the cultivation process. Furthermore, the addition of nutrients can weaken the relationship between host plants and pathogens (Marchetto & Power, 2018), even decreasing the abundance of major soil pathogen groups (Zhang et al., 2023), which might be more pronounced in highly diverse plant communities where pathogen abundance is already low. This could explain why nutrient addition more effectively promotes *R. typhina* growth in communities with higher diversity during intraspecific competition, possibly by reducing pathogen pressure. However, whether the speculation holds true needs to be explored in future research.

We initially expected that in the presence of native plants, high‐diversity plant communities would particularly benefit native plants by diluting pathogens, as the original soil was more likely to contain specialist pathogens harmful to the native plants (Wang, Gao, et al., 2023). However, in communities with higher diversity, the competitive ability of the alien plant *R. typhina* increased significantly, whereas the competitive ability of the native plant *A. altissima* remained unchanged, suggesting that this mechanism cannot fully explain our findings. One possible explanation is that in highly diverse plant communities, symbiotic fungi with strong mutualistic abilities are more common and that these fungi, such as AMF, tend to establish closer associations with *R. typhina*. This may be related to the preferences of AMF for alien plants (Bunn et al., 2015). It is critical to point out that our analysis primarily focused on fungi and did not include bacteria. However, certain bacterial groups also play significant roles in the regulation of competition between native and alien plants (Fahey & Flory, 2021). Investigating whether plant communities of varying diversities can alter resistance to invasion by influencing bacterial communities could be an important future research direction. We further observed that after nutrient addition, the competitive abilities of *R. typhina* and *A. altissima* changed, especially within the context of highly diverse communities. This implies that under conditions of high nutrient availability, the role of AMF can be reduced significantly or even reversed (Chen et al., 2019; Elbon & Whalen, 2015), potentially explaining the phenomena that were observed in the present study.

Given that alien species can evade native specialist pathogens and symbionts, native species invading other regions should exhibit similar traits. Considering the extensive invasion of *A. altissima* in North America, often leading to the replacement of *R. typhina* woodlands (Sladonja et al., 2015), we speculate that the dynamics observed in the experiments conducted in China could be similar in North America, despite differences in environmental backgrounds. In other words, the mutual invasion of *R. typhina* and *A. altissima* may be related to their evasion of specialist pathogens and symbionts; however, the phenomenon could also be influenced by environmental factors such as nutrient availability. This provides us with a perspective to understand the mechanisms behind plant invasion on a global scale.

In summary, microbes may influence the relationship between plant diversity and invasibility, particularly the interactions between hosts and pathogens. Furthermore, symbiotic fungi could also play a significant role in the process. The results of the present study highlight the importance of comprehensively considering the effects of competition mode and soil nutrient status when examining the relationship between plant diversity and invasibility. Future research should explore the relative roles of specialist and generalist pathogens and symbiotic fungi in regulating plant diversity and invasibility.

## 5 POTENTIAL CAVEATS

There are a number of limitations that may have influenced the results and conclusions presented here. First, it is essential to highlight that our examination of the soil fungal community was based on ITS sequencing. Compared with targeted primers specifically for AMF, the ITS sequencing approach might result in underestimation of OTU types and numbers. Therefore, caution is necessary when interpreting insights into the impact of AMF–host interactions on community resistance. Second, although we examined the relationship between microbial‐mediated plant diversity and invasibility, addressing competition and nutrient level, our soil samples were derived from potted plants without direct interactions, such as resource competition. This might introduce biases, as resource competition can affect plant growth and root exudation, thereby altering the microbial community. By mixing soils in which various plant species were grown, we largely eliminated the influence of resource competition, allowing us to focus on the effects of soil microbial diversity. However, to comprehensively understand how resource competition influences the process, future research should include direct interactions between plants.

## AUTHOR CONTRIBUTIONS

**Haokun Li:** Conceptualization (equal); methodology (equal); writing – original draft (lead). **Xinyu Hu:** Conceptualization (equal); methodology (equal). **Xinze Geng:** Methodology (equal). **Bo Xiao:** Methodology (equal). **Wei Miao:** Methodology (equal). **Zhiguang Xu:** Funding acquisition (equal); writing – review and editing (equal). **Yizhuo Deng:** Data curation (equal). **Bohan Jiang:** Data curation (equal). **Yuping Hou:** Funding acquisition (equal); writing – review and editing (equal).

## CONFLICT OF INTEREST STATEMENT

The authors declare that there are no potential conflicts of interest.

## Supporting information


Appendix S1.

## Data Availability

The original data are available in the Dryad data repository: https://doi.org/10.5061/dryad.wh70rxww6. The original data are stored in the Dryad data repository for private peer review: https://datadryad.org/stash/share/wWUaebaQITC5WUXSPWGB743Et5yxBJOvcmDsaGEnsv0.

## References

[ece311425-bib-0001] Abbott, K. C. , Karst, J. , Biederman, L. A. , Borrett, S. R. , Hastings, A. , Walsh, V. , & Bever, J. D. (2015). Spatial heterogeneity in soil microbes alters outcomes of plant competition. PLoS One, 10, e0125788. 25946068 10.1371/journal.pone.0125788 PMC4422530

[ece311425-bib-0002] Barry, K. E. , Mommer, L. , Van Ruijven, J. , Wirth, C. , Wright, A. J. , Bai, Y. , Connolly, J. , De Deyn, G. B. , De Kroon, H. , & Isbell, F. (2019). The future of complementarity: Disentangling causes from consequences. Trends in Ecology & Evolution, 34, 167–180. 30527960 10.1016/j.tree.2018.10.013

[ece311425-bib-0003] Bates, D. M. , Machler, M. , Bolker, B. M. , & Walker, S. C. (2014). Fitting linear mixed‐effects models using lme4. Journal of Statistical Software, 67, 1–48.

[ece311425-bib-0004] Bever, J. D. , Westover, K. M. , & Antonovics, J. (1997). Incorporating the soil community into plant population dynamics: The utility of the feedback approach. Journal of Ecology, 85, 561–573.

[ece311425-bib-0005] Bunn, R. A. , Ramsey, P. W. , & Lekberg, Y. (2015). Do native and invasive plants differ in their interactions with arbuscular mycorrhizal fungi? A meta‐analysis. Journal of Ecology, 103, 1547–1556.

[ece311425-bib-0006] Byers, J. E. , & Noonburg, E. G. (2003). Scale dependent effects of biotic resistance to biological invasion. Ecology, 84, 1428–1433.

[ece311425-bib-0007] Chen, E. , Liao, H. , Chen, B. , & Peng, S. (2019). Arbuscular mycorrhizal fungi are a double‐edged sword in plant invasion controlled by phosphorus concentration. The New Phytologist, 226, 295–300. 10.1111/nph.16359 31808168

[ece311425-bib-0008] Crawford, K. M. , Bauer, J. T. , Comita, L. S. , Eppinga, M. B. , Johnson, D. J. , Mangan, S. A. , Queenborough, S. A. , Strand, A. E. , Suding, K. N. , Umbanhowar, J. A. , & Bever, J. D. (2019). When and where plant‐soil feedback may promote plant coexistence: A meta‐analysis. Ecology Letters, 22, 1274–1284. 31149765 10.1111/ele.13278

[ece311425-bib-0009] Dai, Z. , Wan, L.‐Y. , Qi, S. , Rutherford, S. , Guangqian, R. , Wan, J. S. H. , & Du, D. (2020). Synergy among hypotheses in the invasion process of alien plants: A road map within a timeline. Perspectives in Plant Ecology, Evolution and Systematics, 47, 125575.

[ece311425-bib-0010] Davies, K. F. , Chesson, P. , Harrison, S. P. , Inouye, B. D. , Melbourne, B. A. , & Rice, K. J. (2005). Spatial heterogeneity explains the scale dependence of the native–exotic diversity relationship. Ecology, 86, 1602–1610.

[ece311425-bib-0011] Dong, L.‐J. , Ma, L. , & He, W. (2021). Arbuscular mycorrhizal fungi help explain invasion success of Solidago canadensis. Applied Soil Ecology, 157, 103763.

[ece311425-bib-0012] Elbon, A. , & Whalen, J. K. (2015). Phosphorus supply to vegetable crops from arbuscular mycorrhizal fungi: A review. Biological Agriculture & Horticulture, 31, 73–90.

[ece311425-bib-0013] Elton, C. S. (1958). The ecology of invasions by animals and plants. Springer.

[ece311425-bib-0014] Ernst, A. R. , Barak, R. S. , Hipp, A. L. , Kramer, A. T. , Marx, H. E. , & Larkin, D. J. (2021). The invasion paradox dissolves when using phylogenetic and temporal perspectives. Journal of Ecology, 110, 443–456.

[ece311425-bib-0015] Fahey, C. , & Flory, S. L. (2021). Soil microbes alter competition between native and invasive plants. Journal of Ecology, 110, 404–414.

[ece311425-bib-0016] Fitzpatrick, C. R. , Mustafa, Z. , & Viliunas, J. (2019). Soil microbes alter plant fitness under competition and drought. Journal of Evolutionary Biology, 32, 438–450. 30739360 10.1111/jeb.13426

[ece311425-bib-0017] Frey, K. G. , Herrera‐Galeano, J. , Redden, C. L. , Luu, T. , Servetas, S. L. , Mateczun, A. J. , Mokashi, V. P. , & Bishop‐Lilly, K. A. (2014). Comparison of three next‐generation sequencing platforms for metagenomic sequencing and identification of pathogens in blood. BMC Genomics, 15, 1–14. 24495417 10.1186/1471-2164-15-96 PMC3922542

[ece311425-bib-0018] Fridley, J. D. , Stachowicz, J. J. , Naeem, S. , Sax, D. F. , Seabloom, E. W. , Smith, M. D. , Stohlgren, T. J. , Tilman, D. , & Holle, B. V. (2007). The invasion paradox: Reconciling pattern and process in species invasions. Ecology, 88, 3–17. 17489447 10.1890/0012-9658(2007)88[3:tiprpa]2.0.co;2

[ece311425-bib-0019] Guo, X. , & Xu, Z. (2019). Increased soil moisture aggravated the competitive effects of the invasive tree *Rhus typhina* on the native tree Cotinus coggygria. BMC Ecology, 20, 1–13. 10.1186/s12898-020-00284-9 PMC7106899 32228576

[ece311425-bib-0020] Hart, M. M. , Reader, R. J. , & Klironomos, J. (2003). Plant coexistence mediated by arbuscular mycorrhizal fungi. Trends in Ecology & Evolution, 18, 418–423.

[ece311425-bib-0021] Herben, T. (2005). Species pool size and invasibility of Island communities: A null model of sampling effects. Ecology Letters, 8, 909–917. 34517689 10.1111/j.1461-0248.2005.00790.x

[ece311425-bib-0022] HilleRisLambers, J. , Adler, P. B. , Harpole, W. S. , Levine, J. M. , & Mayfield, M. M. (2012). Rethinking community assembly through the lens of coexistence theory. Annual Review of Ecology, Evolution, and Systematics, 43, 227–248.

[ece311425-bib-0023] Hothorn, T. , Bretz, F. , & Westfall, P. (2008). Simultaneous inference in general parametric models. Biometrical Journal: Journal of Mathematical Methods in Biosciences, 50, 346–363. 10.1002/bimj.200810425 18481363

[ece311425-bib-0024] Hudson, P. J. , Dobson, A. P. , & Lafferty, K. D. (2006). Is a healthy ecosystem one that is rich in parasites? Trends in Ecology & Evolution, 21, 381–385. 16713014 10.1016/j.tree.2006.04.007

[ece311425-bib-0025] Islam, W. , Noman, A. , Naveed, H. , Huang, Z. , & Chen, H. Y. H. (2020). Role of environmental factors in shaping the soil microbiome. Environmental Science and Pollution Research, 27, 41225–41247. 32829437 10.1007/s11356-020-10471-2

[ece311425-bib-0026] Johnson, N. C. (2010). Resource stoichiometry elucidates the structure and function of arbuscular mycorrhizas across scales. The New Phytologist, 185, 631–647. 19968797 10.1111/j.1469-8137.2009.03110.x

[ece311425-bib-0027] Johnson, N. C. , Graham, J. H. , & Smith, F. A. (1997). Functioning of mycorrhizal associations along the mutualism–parasitism continuum. New Phytologist, 135, 575–585.

[ece311425-bib-0028] Kandlikar, G. S. , Johnson, C. A. , Yan, X. , Kraft, N. J. B. , & Levine, J. M. (2019). Winning and losing with microbes: How microbially mediated fitness differences influence plant diversity. Ecology Letters, 22, 1178–1191. 31134744 10.1111/ele.13280

[ece311425-bib-0029] Kaur, S. , Campbell, B. J. , & Suseela, V. (2022). Root metabolome of plant‐arbuscular mycorrhizal symbiosis mirrors the mutualistic or parasitic mycorrhizal phenotype. The New Phytologist, 234, 672–687. 35088406 10.1111/nph.17994

[ece311425-bib-0030] Keesing, F. , Holt, R. D. , & Ostfeld, R. S. (2006). Effects of species diversity on disease risk. Ecology Letters, 9, 485–498. 16623733 10.1111/j.1461-0248.2006.00885.x

[ece311425-bib-0031] Lamit, L. J. , Giovati, A. S. , Jo, I. , Frank, D. A. , & Fridley, J. D. (2022). Woody invaders are more highly colonized by arbuscular mycorrhizal fungi than congeneric native species in a common garden. American Journal of Botany, 109, 655–663. 35266547 10.1002/ajb2.1839

[ece311425-bib-0032] Liao, H. , Luo, W. , Peng, S. , & Callaway, R. M. (2015). Plant diversity, soil biota and resistance to exotic invasion. Diversity and Distributions, 21, 826–835.

[ece311425-bib-0033] Lodge, D. J. (1997). Factors related to diversity of decomposer fungi in tropical forests. Biodiversity and Conservation, 6, 681–688.

[ece311425-bib-0034] Loman, N. J. , Misra, R. V. , Dallman, T. J. , Constantinidou, C. , Gharbia, S. E. , Wain, J. R. , & Pallen, M. J. (2012). Performance comparison of benchtop high‐throughput sequencing platforms. Nature Biotechnology, 30, 434–439. 10.1038/nbt.2198 22522955

[ece311425-bib-0035] Mallon, C. A. , Poly, F. , Le Roux, X. , Marring, I. , Van Elsas, J. D. , & Salles, J. F. (2015). Resource pulses can alleviate the biodiversity‐invasion relationship in soil microbial communities. Ecology, 96, 915–926. 26230013 10.1890/14-1001.1

[ece311425-bib-0036] Marchetto, K. M. , & Power, A. G. (2018). Context‐dependent interactions between pathogens and a mutualist affect pathogen fitness and mutualist benefits to hosts. Ecology, 99, 2833–2843. 30298921 10.1002/ecy.2531

[ece311425-bib-0037] Mitchell, C. E. , & Power, A. G. (2003). Release of invasive plants from fungal and viral pathogens. Nature, 421, 625–627. 12571594 10.1038/nature01317

[ece311425-bib-0038] Morris, W. F. , Hufbauer, R. A. , Agrawal, A. A. , Bever, J. D. , Borowicz, V. A. , Gilbert, G. S. , Maron, J. L. , Mitchell, C. E. , Parker, I. M. , Power, A. G. , Torchin, M. E. , & Vázquez, D. P. (2007). Direct and interactive effects of enemies and mutualists on plant performance: A meta‐analysis. Ecology, 88, 1021–1029. 17536717 10.1890/06-0442

[ece311425-bib-0039] Nguyen, N. H. , Song, Z. , Bates, S. T. , Branco, S. , Tedersoo, L. , Menke, J. R. , Schilling, J. S. , & Kennedy, P. G. (2016). FUNGuild: An open annotation tool for parsing fungal community datasets by ecological guild. Fungal Ecology, 20, 241–248.

[ece311425-bib-0040] Ostfeld, R. S. , & Keesing, F. (2012). Effects of host diversity on infectious disease. Annual Review of Ecology, Evolution, and Systematics, 43, 157–182.

[ece311425-bib-0041] Petruzzellis, F. , Nardini, A. , Savi, T. , Tonet, V. , Castello, M. , & Bacaro, G. (2019). Less safety for more efficiency: Water relations and hydraulics of the invasive tree Ailanthus altissima (mill.) Swingle compared with native Fraxinus ornus L. Tree Physiology, 39, 76–87. 29982793 10.1093/treephys/tpy076

[ece311425-bib-0042] R Core Team . (2021). R: A language and environment for statistical computing. R Foundation for Statistical Computing. http://www.R‐project.org/

[ece311425-bib-0043] Reinhart, K. O. , & Callaway, R. M. (2006). Soil biota and invasive plants. The New Phytologist, 170, 445–457. 16626467 10.1111/j.1469-8137.2006.01715.x

[ece311425-bib-0044] Richardson, D. M. , Allsopp, N. , D'antonio, C. M. , Milton, S. J. , & Rejmánek, M. (2000). Plant invasions–the role of mutualisms. Biological Reviews, 75, 65–93. 10740893 10.1017/s0006323199005435

[ece311425-bib-0045] Richardson, D. M. , & Pyšek, P. (2006). Plant invasions: Merging the concepts of species invasiveness and community invasibility. Progress in Physical Geography, 30, 409–431.

[ece311425-bib-0046] Schmidt, K. A. , & Ostfeld, R. S. (2001). Biodiversity and the dilution effect in disease ecology. Ecology, 82, 609–619.

[ece311425-bib-0047] Seebens, H. , Blackburn, T. M. , Dyer, E. E. , Genovesi, P. , Hulme, P. E. , Jeschke, J. M. , Pagad, S. , Pyšek, P. , Winter, M. , Arianoutsou, M. , Bacher, S. , Blasius, B. , Brundu, G. , Capinha, C. , Celesti‐Grapow, L. , Dawson, W. , Dullinger, S. , Fuentes, N. , Jäger, H. , … Essl, F. (2017). No saturation in the accumulation of alien species worldwide. Nature Communications, 8, 14435. 10.1038/ncomms14435 PMC5316856 28198420

[ece311425-bib-0048] Sladonja, B. , Sušek, M. , & Guillermic, J. (2015). Review on invasive tree of heaven (Ailanthus altissima (mill.) Swingle) conflicting values: Assessment of its ecosystem services and potential biological threat. Environmental Management, 56, 1009–1034. 26071766 10.1007/s00267-015-0546-5

[ece311425-bib-0049] Smith, N. S. , & Côté, I. M. (2019). Multiple drivers of contrasting diversity‐invasibility relationships at fine spatial grains. Ecology, 100(2), e02573. 30516274 10.1002/ecy.2573

[ece311425-bib-0050] Stein, C. , & Mangan, S. A. (2020). Soil biota increase the likelihood for coexistence among competing plant species. Ecology, 101, e03147. 33460105 10.1002/ecy.3147

[ece311425-bib-0051] Sun, D. , Yang, X. , Wang, Y. , Fan, Y. , Ding, P. , Song, X. E. , Yuan, X. , & Yang, X. (2022). Stronger mutualistic interactions with arbuscular mycorrhizal fungi help Asteraceae invaders outcompete the phylogenetically related natives. The New Phytologist, 236, 1487–1496. 35975696 10.1111/nph.18435

[ece311425-bib-0052] Waldrop, M. P. , Zak, D. R. , Blackwood, C. B. , Curtis, C. D. , & Tilman, D. (2006). Resource availability controls fungal diversity across a plant diversity gradient. Ecology Letters, 9, 1127–1135. 16972876 10.1111/j.1461-0248.2006.00965.x

[ece311425-bib-0053] Wang, G. , Burrill, H. M. , Podzikowski, L. Y. , Eppinga, M. B. , Zhang, F. , Zhang, J. , Schultz, P. A. , & Bever, J. D. (2023). Dilution of specialist pathogens drives productivity benefits from diversity in plant mixtures. Nature Communications, 14, 8417. 10.1038/s41467-023-44253-4 PMC10728191 38110413

[ece311425-bib-0054] Wang, G. , Jiang, G.‐M. , Yu, S. , Li, Y. , & Liu, H. (2008). Invasion possibility and potential effects of *Rhus typhina* on Beijing municipality. Journal of Integrative Plant Biology, 50, 522–530. 18713419 10.1111/j.1744-7909.2008.00660.x

[ece311425-bib-0055] Wang, R. , & Zhou, G. (2000). Vegetation of Shandong. Shandong Science and Technology Press.

[ece311425-bib-0056] Wang, X.‐Y. , Gao, S. , Chen, T. , Wang, J. , & Yu, F. (2022). Interactions between soil microbes and native species drive a diversity‐invasibility relationship. Biological Invasions, 25, 1461–1472.

[ece311425-bib-0057] Wang, X.‐Y. , Gao, S. , Chen, T. , Wang, J. , & Yu, F.‐H. (2023). Interactions between soil microbes and native species drive a diversity‐invasibility relationship. Biological Invasions, 25, 1461–1472.

[ece311425-bib-0058] Yang, G. , Yang, X. , Zhang, W. , Wei, Y. , Ge, G. , Lu, W. , Sun, J. , Liu, N. , Kan, H. , Shen, Y. , & Zhang, Y. (2016). Arbuscular mycorrhizal fungi affect plant community structure under various nutrient conditions and stabilize the community productivity. Oikos, 125, 576–585.

[ece311425-bib-0059] Yu, W. (2009). Value of ecosystem services of Kunyu Mountain natural reserve. Acta Ecologica Sinica, 29, 523–531.

[ece311425-bib-0060] Zhang, X. , Van Kleunen, M. , Chang, C. , & Liu, Y. (2023). Soil microbes mediate the effects of resource variability on plant invasion. Ecology, 104, e4154. 37611168 10.1002/ecy.4154

[ece311425-bib-0061] Zhang, Z. , Liu, Y. , Brunel, C. , & Van Kleunen, M. (2020). Evidence for Elton's diversity‐invasibility hypothesis from belowground. Ecology, 101, e03187. 32893873 10.1002/ecy.3187

[ece311425-bib-0062] Zhang, Z. , Pan, X. , Blumenthal, D. , Van Kleunen, M. , Liu, M. , & Li, B. (2018). Contrasting effects of specialist and generalist herbivores on resistance evolution in invasive plants. Ecology, 99, 866–875. 29352479 10.1002/ecy.2155

