# Supplementary material for: Competition mode and soil nutrient status shape the role of soil microbes in the diversity–invasibility relationship
Source: Ecol Evol. 2024 May 14;14(5):e11425. doi: 10.1002/ece3.11425 (PMC11091549; doi:10.1002/ece3.11425)
Supplement: Supplementary file 1 — Appendix S1. [file ECE3-14-e11425-s001.docx]

**Supplementary Information**

**Competition mode and soil nutrient status shape the role of soil microbes in the diversity–invasibility relationship**

Haokun Li, Xin Hu, Xinze Geng, Zhiguang Xu, Bo Xiao, Wei Miao, Yizhuo Deng, Bohan Jiang, and Yuping Hou

**FIGURE S1**
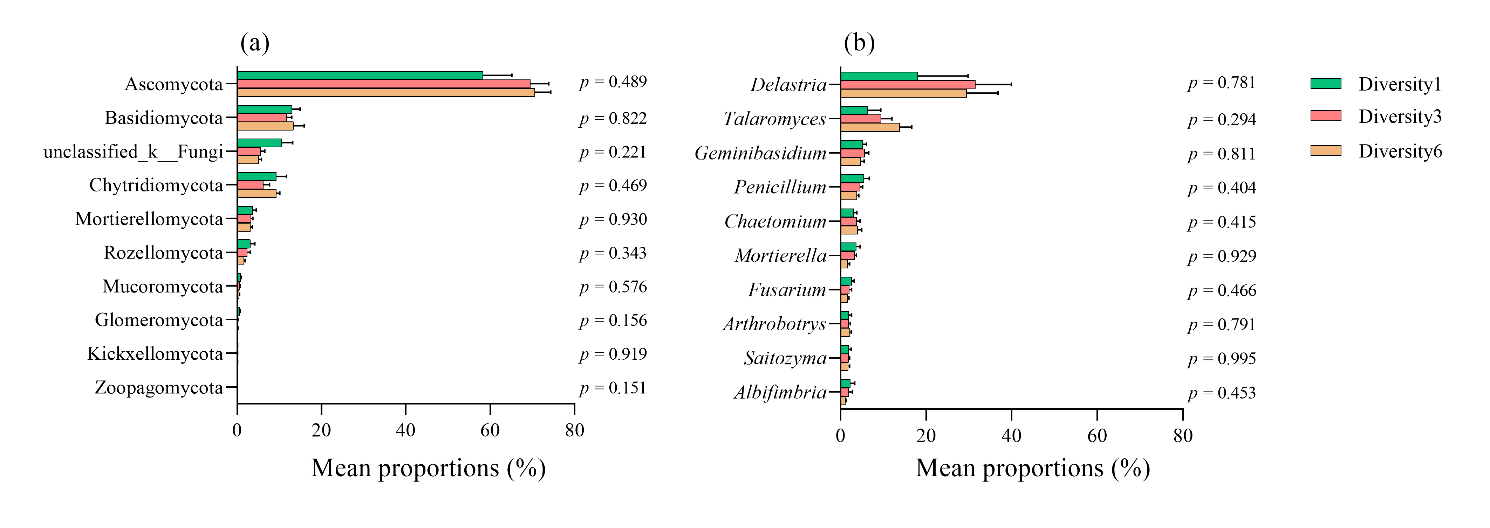


FIGURE S1. Relative abundances of dominant fungal phyla and genera present in soils conditioned with different levels of plant diversity. (a) Relative abundance of the dominant fungal phyla. (b) Relative abundance of dominant fungal genera. None of the differences are significant at *p* < 0.05.

**FIGURE S2**


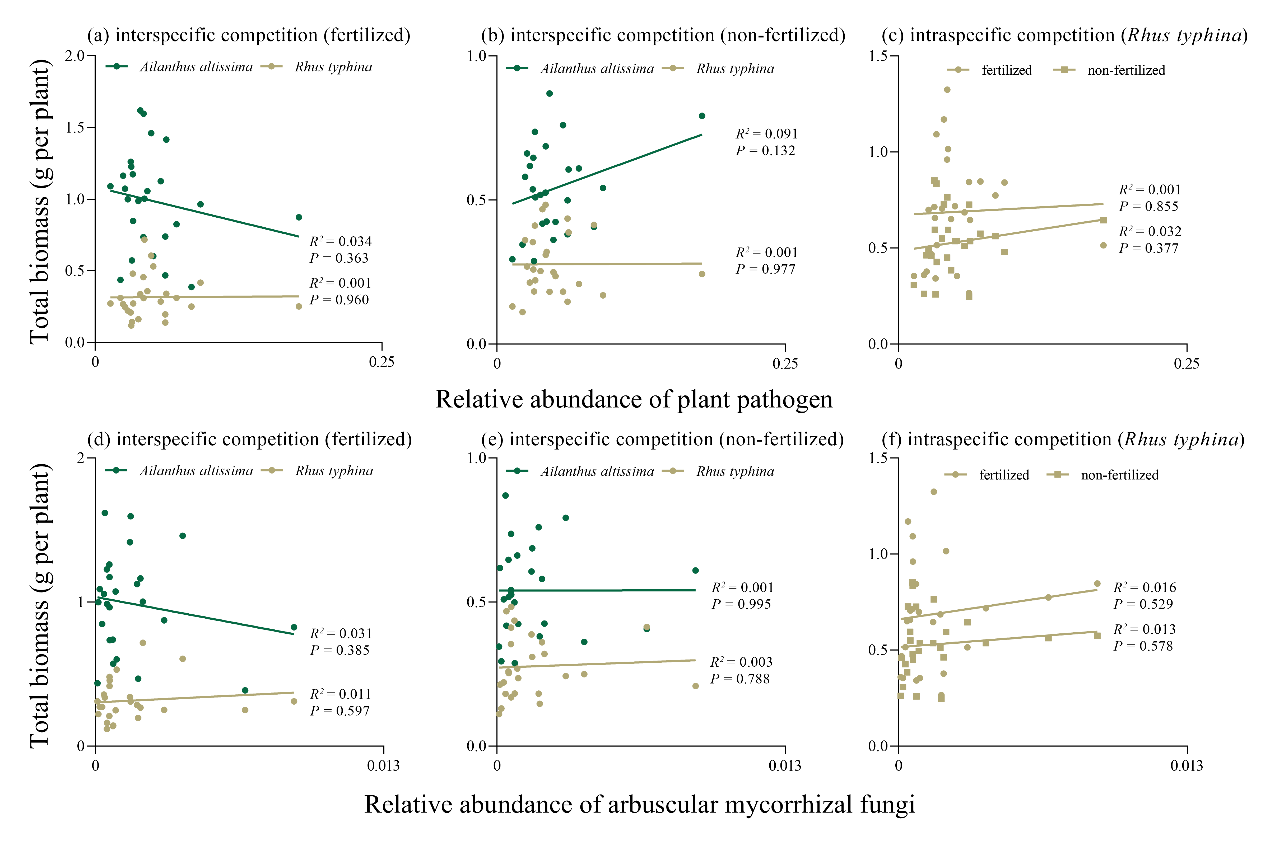


FIGURE S2. Shannon index of arbuscular mycorrhizal fungi and plant pathogens in relation to total biomass per plant for *Rhus typhina* and *Ailanthus altissima* under different competition modes (interspecific and intraspecific) and nutrient levels (fertilized and non-fertilized). (a–c) Relative abundance of plant pathogens and total biomass per plant for *R. typhina* and *A. altissima*. (d–f) Relative abundance of arbuscular mycorrhizal fungi and total biomass per plant for *R. typhina* and *A. altissima*.

**TABLE S1** Chinese native species used in this study

| Species | Family | Plant form |
| --- | --- | --- |
| *Indigofera kirilowii* Maxim. ex Palib. | Fabaceae | Deciduous shrub |
| *Grewiabiloba var. parviflora* (Bunge)Hand.-Maz. | Malvaceae | Deciduous shrub |
| (Bunge)Hand. -Maz. |  |  |
| *Lespedeza bicolor* Tucz. | Fabaceae | Deciduous shrub |
| *Rhus chinensis* Mill. | Anacardiaceae | Deciduous shrub |
| *Smilax china* L. | Smilacaceae | Climbing vine |
| *Pinus densiflora* Sieb. et Zucc. | Pinaceae | Evergreen Conifer |
| *Ailanthus altissima* (Mill.) Swingle | Simaroubaceae | Deciduous tree |
| *Quercus acutissima* Carr. | Fagaceae | Deciduous tree |
| *Quercus variabilis* Blume. | Fagaceae | Deciduous tree |

**TABLE S2** Effect of plant diversity on alien plants for 27 soil mixture types

|  | Diversity1 | | | | | | | | | Diversity3 | | | | | | | | | Diversity6 | | | | | | | | |
| --- | --- | --- | --- | --- | --- | --- | --- | --- | --- | --- | --- | --- | --- | --- | --- | --- | --- | --- | --- | --- | --- | --- | --- | --- | --- | --- | --- |
| Community | 1 | 2 | 3 | 4 | 5 | 6 | 7 | 8 | 9 | 10 | 11 | 12 | 13 | 14 | 15 | 16 | 17 | 18 | 19 | 20 | 21 | 22 | 23 | 24 | 25 | 26 | 27 |
| *Indigofera kirilowii* |  |  |  |  |  |  |  |  |  |  |  |  |  |  |  |  |  |  |  |  |  |  |  |  |  |  |  |
| *Grewia biloba* |  |  |  |  |  |  |  |  |  |  |  |  |  |  |  |  |  |  |  |  |  |  |  |  |  |  |  |
| *Lespedeza bicolor* |  |  |  |  |  |  |  |  |  |  |  |  |  |  |  |  |  |  |  |  |  |  |  |  |  |  |  |
| *Rhus chinensis* |  |  |  |  |  |  |  |  |  |  |  |  |  |  |  |  |  |  |  |  |  |  |  |  |  |  |  |
| *Smilax china* |  |  |  |  |  |  |  |  |  |  |  |  |  |  |  |  |  |  |  |  |  |  |  |  |  |  |  |
| *Pinus densiflora* |  |  |  |  |  |  |  |  |  |  |  |  |  |  |  |  |  |  |  |  |  |  |  |  |  |  |  |
| *Ailanthus altissima* |  |  |  |  |  |  |  |  |  |  |  |  |  |  |  |  |  |  |  |  |  |  |  |  |  |  |  |
| *Quercus acutissima* |  |  |  |  |  |  |  |  |  |  |  |  |  |  |  |  |  |  |  |  |  |  |  |  |  |  |  |
| *Quercus variabilis* |  |  |  |  |  |  |  |  |  |  |  |  |  |  |  |  |  |  |  |  |  |  |  |  |  |  |  |

Each column represents one type of soil mixture. Yellow cells indicate the species used to condition each soil mixture. Diversity1: Soils conditioned using one species; Diversity3: Soils conditioned using three species; Diversity6: Soils conditioned using six species. Each species contributed to one mixture for Diversity1, three mixtures for Diversity3, and six mixtures for Diversity6.

**TABLE S3** Effects of plant diversity (Div), nutrient status (Nutr), and their interactive effects on square root-transformed total *Ailanthus altissima* biomass

| **Factors/**  **interactions** | **Total biomass of *Ailanthus altissima* (square root-transformed)** | | |
| --- | --- | --- | --- |
|  | *F* | *df* | *P* |
| Div | 2.321 | 2 | 0.120 |
| Nutr | 58.12 | 1 | **<0.01** |
| Div×Nutr | 4.765 | 2 | **<0.01** |

Differences are considered significant at *p* < 0.05 and marginally significant at *p* < 0.1.
